# The Selective Dopamine D2 Blocker Sulpiride Modulates the Relationship Between Agentic Extraversion and Executive Functions

**DOI:** 10.3758/s13415-021-00887-9

**Published:** 2021-04-02

**Authors:** Wiebke Herrmann, Jan Wacker

**Affiliations:** Department of Psychology, University of Hamburg, Von-Melle-Park 5, Room 4020, D-20146 Hamburg, Germany

**Keywords:** Cognitive control, Dopamine, Extraversion, Executive functions

## Abstract

Initial studies suggest that agentic extraversion and executive functions (EF) are associated, because they share influences of individual differences in the dopamine (DA) system. However, it is unclear whether previously reported associations are specific to certain EFs (e.g., to updating or shifting) or due to shared variance among EF tasks. We investigated the DA-related relationship between agentic extraversion and two EF tasks in a placebo-controlled between-group design with the DA D2 receptor blocker sulpiride (200 mg) in 92 female volunteers. Our goals were to investigate whether (1) there is an association between agentic extraversion and EFs measured with two different tasks (3-back and switching), (2) this association is sensitive to a pharmacological manipulation of DA, and (3) the effects can be ascribed to shared or specific task variance. We observed the expected interaction between drug condition and agentic extraversion for both tasks in a multivariate multiple linear regression model, which supports the DA theory of extraversion. Subsequent univariate analyses revealed a highly similar interaction effect between drug condition and agentic extraversion on two of three performance measures and this effect was somewhat attenuated when we controlled for shared task variance. This pattern matches the interpretation that the association between agentic extraversion and both tasks is partly due to DA-based processes shared among the tasks. Our results, although limited by the low reliability of the switching task, suggest that variance components and measurement difficulties of EF tasks should be considered when investigating personality-related individual differences in EFs.

**Supplementary Information:**

The online version contains supplementary material available at 10.3758/s13415-021-00887-9.

## Introduction

According to a prominent psychobiological theory of extraversion, individual differences in extraversion, especially in its agentic component comprising reward responsiveness, assertiveness, activity, drive, and ambitiousness, are partly driven by individual differences in dopaminergic reward and incentive processing (Depue & Collins, 1999). Dopamine has been associated with several other processes, including executive functions (EFs), such as working memory updating (Luciana et al., 1992) and shifting (Fallon et al., 2015). Initial studies suggest that individual differences in these EFs might likewise be associated with extraversion (Lieberman & Rosenthal, 2001), prompting the idea of an overlap in the underlying dopaminergic mechanisms. However, the overall number of studies on the relationship between (agentic) extraversion and EFs is limited, and we are not aware of any studies on the dopaminergic relationship between extraversion and EFs employing more than one EF task within the same study. This constitutes a significant limitation, because—as we will review in more detail in the next section—EF tasks are known to not only target one isolated mechanism. Systematic performance variance in EF tasks is comprised of (1) variance shared by all EF tasks, (2) variance only shared by tasks targeting a specific EF, (3) variance not related to any EF but to other cognitive processes the task recruits (e.g. processing of colors, numbers, or faces). On a neural level, these different sources of performance variance are reflected in a complex system of several interacting areas, including the prefrontal cortex, parietal cortex, and the basal ganglia (Friedman & Miyake, 2017).

The measurement difficulties of EFs caused by the several variance components in EF task performance are often addressed with a simultaneous analysis of performance in several EF tasks, e.g., with latent variable analysis, in order to differentiate variance components (Friedman & Miyake, 2017). This quite time-intensive approach seems less common for the investigation of third-variable associations, although one EF task alone cannot separate individual differences within each of the variance components of task performance. It is therefore possible that previous studies overestimated the specificity of reported associations between agentic extraversion and EF task performance, which could not only be attributable to the targeted particular EFs (i.e., updating and shifting) but also to other systematic variance components. For the investigation of a relationship between agentic extraversion and EFs, it is necessary to take shared task-variance into consideration.

### Executive Functions

Despite a large body of relevant research, the definition and measurement of specific EFs still is a challenging task (Miyake & Friedman, 2012). EFs are understood as top-down control mechanisms that regulate the dynamics of human cognition and action. These mechanisms substantially correlate but also seem to tap into distinct mechanisms, being described as showing both “unity” and “diversity.” Further measurement difficulties arise from the fact that EF tasks must operate on a specific task context (e.g., processing of faces, colors or letters) and therefore necessarily include systematic variance not related to the targeted EF—a phenomenon termed “task impurity.” In order to at least partly cancel out task impurity, EFs often are measured with several tasks operating on different task contexts so that their shared variance can be extracted in a latent variable approach (Miyake & Friedman, 2012). The resulting latent EF variables will still correlate substantially, which demonstrates that performance in EF tasks is not only comprised of variance specific to the EF that was targeted (diversity) but also of shared variance among all EF tasks (unity).

The diversity of EFs also is reflected in differential third-variable associations between specific EFs and for example intelligence (Friedman et al., 2006), which can even vary in direction once the unity of EFs is partialed out—a principle that also could apply to the previously reported associations with agentic extraversion. Because these theoretically could be due to several systematic variance components, a consideration of these components is necessary to draw conclusions about specific relationships, e.g., between agentic extraversion and shifting (Berse et al., 2014). We are currently aware of only one study that indicates that extraversion-related performance differences can be ascribed to EF-processes, and not just task impurity, by computing mean scores across several EF tasks operating on various task contexts (e.g., colors, geometric shapes, words, affective categories; Campbell et al., 2011). Performance differences were found for updating and shifting tasks, which we will review in the next sections.

#### Updating

Among the numerous EFs, *updating* is probably the one most often associated with extraversion. EF tasks which target updating require participants to monitor, retrieve, transform, and substitute working memory content (Ecker et al., 2010; Miyake & Friedman, 2012). The two candidate mechanisms specific to the concept of updating are effective gating of information and controlled retrieval from long-term memory (Miyake & Friedman, 2012). The n-back task is a common task in the context of EFs. It is mostly known as a measure for updating because it requires a consecutive buffering of continually changing information (Miyake et al., 2000; Smith & Jonides, 1997). While the 1-back version of the task simply requires participants to decide whether one letter is identical to the one presented directly before, the 2- and 3-back versions are more demanding and draw on several executive processes, like the active maintenance of relevant items and resistance to proactive interference from currently irrelevant items (Chatham et al., 2011). Performance differences associated with the agentic aspect of extraversion only show up in the more complex versions of the n-back task (Gray & Braver, 2002; Lieberman & Rosenthal, 2001; Wacker et al., 2006), indicating that agentic extraversion potentially can be associated with working memory processes rather than the short-term memory processes involved in the 1-back version. A similar pattern was found in another study, where (agentic) extraversion-related performance differences in updating tasks also only occurred in difficult task versions, albeit with different EF tasks (Campbell et al., 2011). Taken together, emerging evidence suggests an association between agentic extraversion and updating.

#### Shifting

Another EF with a potential association with agentic extraversion is *shifting*. This function reflects processes which direct the attentional focus towards new goals or task-sets. We consider shifting to be synonymous with *switching* and understand both as process-oriented terms tied to the broader concept of *cognitive flexibility.*^1^ Cognitive flexibility opposes cognitive stability, that is, the active maintenance of current goals or task-sets (Cools & D’Esposito, 2011; Monsell, 2003). For functional behavior, it is important to find a balance between actively maintaining current information and directing the attentional focus towards new information, which also is known as the stability-flexibility-dilemma (Dreisbach, 2006; Jongkees & Colzato, 2016). There is ongoing debate about the definition of EFs, especially regarding the broad concept of cognitive flexibility (Boot et al., 2017). Even the definitions of the rather process-oriented terms updating and shifting seem to share the idea that they entail processes that adaptively update current working memory representations. Kessler et al. (2017) even argue that shifting is a special case of working memory updating and that both functions rely mostly on the same processes that remove irrelevant representations from working memory and update it with newly relevant representations. If this were the case, previously reported third-variable associations between agentic extraversion and either EF might be attributable to the same underlying mechanism(s) (Kessler et al., 2017).

Many set-shifting tasks analyze rapid, frequent switches between two task sets as a measure of flexibility without dissociating the functional advantages and disadvantages of high versus low flexibility. They usually follow the pattern that participants are asked to react to certain stimulus features while ignoring other features (e.g., colors, categories, odd/even numbers). A *switch* between the features participants are asked to focus on results in reaction time costs depending on the strength of participants’ prior task-set representations and their ease of transitioning to the new task-set. In every nonswitch trial of the task, participants’ performance is optimal if they are able to actively maintain the representation of the current task-set. When a switch becomes necessary because the attentional focus needs to be shifted towards a new task-set (Friedman & Miyake, 2017), costs in reaction times might occur because the focus on the preswitch task hinders disengagement even after the switch. This perseverative behavior would be a sign of relatively low flexibility and might be caused by a high threshold in updating (vs. maintaining) task-set representations. However, switch costs might not only occur because the updating threshold is too high, but also when it is too low. Whereas a low threshold is beneficial for adaptively updating task-set representations, it also comes with a weaker active maintenance of the correct task-set representation, increasing the risk for distraction by irrelevant information. This distractibility would be a sign of too high flexibility and might become even more apparent when the distracting information prompts more attention, because it is new and needs a closer examination (Dreisbach, 2006; Dreisbach & Fröber, 2019; Dreisbach & Goschke, 2004; Goschke & Bolte, 2014).

The *switching task* developed by Dreisbach and Goschke (2004) aims to dissociate the costs and benefits of a bias for updating versus maintaining a task-set with the help of two different switching conditions. These conditions address the described balance between distractibility, hence too active updating of working memory representations, versus perseveration, hence too active maintenance of working memory representations. Although this study cannot provide a solution for the conceptual unclarities regarding updating and shifting, a differentiation between two possible mechanisms behind higher switch costs might be helpful to explain the mixed results of prior studies reporting either a negative relationship between agentic extraversion and cognitive flexibility (Wacker, 2018), a positive relationship (Berse et al., 2014), or no relationship (Murdock et al., 2013; Vaughan & Edwards, 2020). Although the results of prior studies are mixed at best, they are all based on the same idea that a potential association between agentic extraversion and cognitive flexibility might be due to a partly shared dopaminergic regulation.

### Neural Mechanisms

EF tasks activate a large, integrated neural network including frontal, cingulate, parietal, and subcortical regions (Niendam et al., 2012). Most elements of this network are shared across most EF tasks due to their unitary function for high-level processing (e.g., frontoparietal network; Reineberg et al., 2018), while some elements are process-specific due to their modular, specialized function only necessary either for certain specific EFs (e.g., lateral PFC regions, amongst others, for shifting attention towards a new goal; Lemire-Rodger et al., 2019) or for processing particular representations of task characteristics (e.g., fusiform face area for face processing; Kanwisher et al., 1997). According to computational models as well as clinical studies, the striatum plays a key role in this neural network by performing a gating function. Via its projections to the cortex within the corticostriatal loop (Doll & Frank, 2009), the striatum regulates the updating of current working memory representations in the prefrontal cortex (Chiew & Braver, 2017; Doll & Frank, 2009). Striatal and prefrontal DA create a dynamic balance, with increased DA levels in the striatum associated with decreased DA levels in the prefrontal cortex, and vice versa (Cools & D’Esposito, 2011). This balance affects the updating versus maintenance of working memory representations, with a DA striatal loop regulating the updating of representations via phasic DA release, and a DA prefrontal cortical loop stabilizing representations via tonic DA release (Cools, 2016; Yee & Braver, 2018).

The balance between striatal and prefrontal DA can be affected by pharmacological manipulation of either of their components, with the direction of effects depending on functionally and regionally specific pharmacological effects (Cools & D’Esposito, 2011). For instance, performance after intake of the DA D2 antagonist sulpiride, which affects DA signaling in the striatum (Sigala et al., 1991), has been shown to depend on striatal DA synthesis capacity (Westbrook et al., 2020). This demonstrates that a dopaminergic drug which affects striatal DA activation can improve shifting performance for individuals with low baseline performance and, conversely, decrease it for individuals with high baseline performance (Cools et al., 2007; Cools & D’Esposito, 2011; Kimberg et al., 1997). Therefore, rather than being linearly associated, striatal DA activation and performance are linked via an inverted U-shaped function.

Extraversion has been associated with individual differences in striatal DA receptor density (Baik et al., 2012) and gray matter volume in the caudate and nucleus accumbens (Lai et al., 2019; Li et al., 2019). A differential reaction to dopaminergic drugs, visible in extraversion-related changes in performance, i.e., performance increments for introverts and performance decrements for extraverts, might therefore be indicative of individual differences in baseline DA. In other words, a potential association between (agentic) extraversion and EF performance, which is sensitive to a pharmacological manipulation of sulpiride, would indicate an overlap in the underlying dopaminergic mechanisms in the striatum.

Investigating the effects of a pharmacological manipulation of DA on more than one EF task within the same study therefore is not only a fruitful approach to differentiate specific and shared EF processes, but also for investigating the potential dopaminergic overlap of EFs and agentic extraversion to further elucidate extraversion’s dopaminergic basis.

### The Current Research

Taken together, so far, there is initial evidence for an association between agentic extraversion and performance in both n-back tasks and tasks targeting cognitive flexibility. Both associations have been, theoretically and/or experimentally, connected with individual differences in DA: Individual differences in reward/incentive salience processing thought to partly underlie both trait extraversion (Depue & Collins, 1999; Li et al., 2019), and performance differences in the n-back and cognitive flexibility tasks (Berse et al., 2014; Lieberman & Rosenthal, 2001; Wacker, 2018; Wacker et al., 2006) might partly result from shared (dopaminergic) mechanisms. However, all EF tasks rely on a large, integrated neural network and simultaneously tap into several EF and non-EF processes. Because none of the previous studies linking extraversion and EFs have used several tasks to differentiate the unity and diversity of EFs and/or the task impurity problem, it is currently unclear whether we are dealing with several coexisting, specific agentic extraversion-EF associations that also may be due to separable biological sources of variance, or alternatively, with a more general association between agentic extraversion and a unitary component of EF variance common to most EF tasks. Therefore, to extend previous findings on the presumably DA-based relationship between agentic extraversion and EFs, we investigated the association between agentic extraversion and the performance in two EF tasks (3-back letter task, and a color-switching-task with letters and numbers) after administration of either placebo or the selective DA D2 receptor blocker sulpiride.

More specifically, the aims of the current study were to investigate (1) whether there is a relationship between agentic extraversion and EFs measured with either the 3-back and/or the switching task, (2) whether this relationship is sensitive to a manipulation of brain DA, and (3) whether the effects are due to shared or specific task variance in the EF tasks. We expected to find a significant interaction effect between agentic extraversion and condition (sulpiride vs. placebo) on EF task performance in a multivariate model. We further expected to find the same significant interaction effect in univariate models for each of the dependent variables. When controlling for the respective other dependent variables, the effects could either (1) be attenuated or disappear, suggesting that they are (partly) due to shared task variance, or (2) remain of the same magnitude as before, suggesting that they are task-specific to the switching- or to the 3-back task.

## Methods

### Participants

We analyzed a sample of 92 healthy female volunteers (mean age = 22.6, *SD* = 2.5, range 18-31 years, German natives) who participated in this study in exchange for a financial compensation (€55-€65). This study was part of a larger research project on the neural foundations of personality and emotion (further results can be found in Burgdorf et al., 2015; Mueller, Burgdorf, Chavanon, Schweiger, Hennig, et al., 2014; Mueller, Burgdorf, Chavanon, Schweiger, Wacker, & Stemmler, 2014; Schweiger et al., 2013; Wacker, 2018). A post-hoc sensitivity analysis for a MANOVA with two groups (sulpiride vs. placebo), one predictor (agentic extraversion) and three response variables in G*Power 3 demonstrated that a sample size of *N* = 92 (and analysis-*n* = 82) was sufficient to detect a small to medium effect size of *f*^2^ = 0.12 (for analysis-*n*: *f*^2^ = 0.14) with an alpha of 0.05 and a power of 0.80 (Faul et al., 2007). After being recruited on campus, participants came to the lab for a pretesting to check if they met all inclusion criteria (body mass index ≥ 17.5, blood pressure > 90/50, right-handed, unmedicated except for hormonal contraception), and none of the exclusion criteria (self-reported physical impairment, pregnancy, habitual smoking, habitual abuse of drugs or alcohol, psychological disorders now or in the past [assessed with a standardized clinical interview]). All participants reported to be in a romantic heterosexual relationship, which was necessary for a study part reported in Burgdorf et al. (2015). The study protocol was approved by the ethics committee of the German Psychological Society (DGPs).

### Manipulation

Participants received either a capsule with 200 mg of the DA D2 receptor blocker sulpiride or a nondistinguishable placebo for oral consumption in a randomized, double-blind between-subjects design. Although sulpiride is a DA blocker, low dosages of sulpiride (50-300 mg) are reported to have DA-increasing effects due to a dose-related overbalance of its binding to presynaptic DA autoreceptors (vs. postsynaptic DA receptors in higher dosages; Mauri et al., 1996; Kuroki et al., 1999).

### Questionnaires and Tests

Participants completed the German version of the NEO-PI-R (Ostendorf & Angleitner, 2004). Additionally, participants’ romantic partners provided a third-person rating with the respective version of the NEO-PI-R. The two ratings were combined into an average rating of each participants’ personality. The self- and partner ratings for all NEO facets correlated significantly (all *r*s > 0.23, all *p*s < 0.04), with extraversion displaying the highest correlation (*r*(80) = 0.60, *p* < 0.001). Due to experimenter error/equipment failure, two participants had either a self- or a partner rating (but not both), so that we only used the available version instead of the average of the self- and partner rating. The scores for NEO agentic extraversion were calculated as in prior work as mean scores for the NEO extraversion facets assertiveness and activity (Wacker, 2018). The scores for NEO affiliative extraversion were calculated as mean scores for the NEO facets warmth and gregariousness. Participants also completed the Multidimensional Personality Questionnaire (MPQ; Tellegen & Waller, 2008). The mean score of the z-standardized MPQ scales social potency and achievement was used as an alternative measure for agentic extraversion (Morrone-Strupinsky & Depue, 2004). Participants further completed Cattell’s Culture Fair Intelligence Test (CFT 3; Cattell & Weiß, 1971), and several other questionnaires which were of interest for other parts of the project.

### Dependent Variables

#### 3-back Task

This task was the exact same version as the 3-back version used by Wacker et al. (2006). Participants were presented 180 trials in total, of which the first 60 trials were practice trials used to determine individual reaction time criteria for response feedback (see below), which were excluded from the analysis. Every trial consisted of one white letter on a black screen presented for 500 ms, followed by a pause of 1,650 ms. Participants were instructed to indicate via mouse button press (left button for *yes*, right button for *no*) whether the presented letter was identical to the letter that was presented three trials before and to respond as fast and accurately as possible. Participants received a standardized verbal feedback (350 ms) on whether their response was *correct*, *incorrect,* or *slow* after each trial to penalize both errors and slow responding (sound files were comparable in length and volume), because verbal task instructions alone may elicit variation in response criteria within and between subjects, causing additional error variance in a potential speed-accuracy-tradeoff (Heitz, 2014). Slow reaction times were defined by being below the 90^th^ percentile of a participant’s reaction time distribution in the last 50 practice trials (Wacker et al., 2006). Participants did not receive a *slow* feedback during practice trials. Of 120 trials, only the last 117 trials were evaluated, because the first three letters could, by definition, not be classified as targets. Among the evaluated trials, participants were presented 40 target trials, 65 nontarget trials, and 12 lure trials (1-back and 2-back) in a fixed random order. Lure trials were implemented to elicit top-down behavioral adjustments, prioritizing the recollection of items over responding based on their familiarity (Szmalec et al., 2011).

For our main analysis, we calculated the percentage of correct reactions in target trials (accuracy), as well as mean reaction times (speed) for correct target trials as performance measures. We decided to focus on performance in target trials in order to make our results comparable to a prior study on extraversion effects in the 3-back task (Wacker et al., 2006). A statistical analysis of other performance indices, such as RT variability, discrimination index d’, and response bias C will be reported in the Supplement.

#### Switching Task

We presented six blocks of 60 trials which either contained pairs of letters (A, E, O, U, K, M, R, or S) or numbers (2, 3, 4, 5, 6, 7, 8, or 9) in alternating order, and in varying colors (Müller, Dreisbach, Goschke, et al., 2007). Within each block, one color was constantly set as the target-color and another color as the distractor-color.

For each pair of letters/numbers, one was colored as the target and one as the distractor. Participants were instructed to ignore the distractor and to indicate via button press whether the target was a consonant or a vowel (or an even or uneven number) with the left mouse button representing consonants and even numbers, and the right mouse button representing vowels and uneven numbers. Every trial started with two letters (or numbers) presented above and below the fixation cross until participants gave their response. Correct trials were followed by a pause of 1,000 ms, whereas incorrect trials were followed by a pause of 2,000 ms. After 40 trials of each 60-trial-block, participants were informed via a message on the screen that the target-color will *switch* for the remaining trials (e.g., “Change to red”). This color change happened in two different ways (3 blocks each in alternating order): In the condition *learned irrelevance*, the prior distractor-color now became the target color, and a new color was used as distractor-color. In the condition *perseveration*, the prior target-color now became the distractor-color, and a new color was used as target-color. Participants were instructed to respond as fast as possible while avoiding mistakes. The task started with 20 practice trials in which participants gave responses to the target-letters and numbers without distractors.

The switching task is not as cognitively demanding as the 3-back task, which is visible in low error rates (i.e. 3.2% in Müller, Dreisbach, Brocke, et al., 2007) and little variance in accuracy, especially around the actual switch within each block. Performance differences are therefore mostly reflected in reaction times, which is why we measured task performance with a summary index previously reported for this switching task as a measure of the degree or cognitive flexibility relative to stability (Müller, Dreisbach, Goschke, et al., 2007). For this index, we first computed switch costs as the increase in mean reaction times for the five correct trials directly before versus five correct trials directly after the switch (trial 36-40 and trial 41-45), matching the approach in prior work. Because the previously employed fixed number of five trials around the switch is somewhat arbitrary, we also calculated switch costs for a larger interval around the switch (10 correct trials) as an alternative measure, which is in Table 2 in the Supplement. We then calculated a difference score of mean switch costs in the learned irrelevance condition minus mean switch costs in the perseveration condition.

The switch cost difference is a suitable summary index for individual differences in cognitive flexibility, because it taps into both the costs and benefits of a high versus low updating threshold (Müller, Dreisbach, Brocke, et al., 2007; Müller, Dreisbach, Goschke, et al., 2007). Increased flexibility, hence a low updating threshold, should facilitate the disengagement from prior targets, which is thought to be further supported by a bias towards novel stimuli (Dreisbach et al., 2005). This should be especially beneficial in the perseveration condition because of two mechanisms: 1) a low updating threshold, and therefore a lower “stickiness” of the irrelevant cognitive representation of the previous target color (Herd et al., 2014), should facilitate the disengagement from the previous target color, which becomes the distractor color after the switch. Second, the target color after the switch is a new color, which might be more easily updated with a stronger bias towards novel stimuli. Increased stability; hence a high updating threshold, should in contrast facilitate the focus on the preswitch target by shielding it from interference. Performance is disturbed after the switch when now irrelevant, but “sticky” representations are not cleared out fast enough. Additionally, the higher updating threshold should slow down the transition to the new target color, because new information is not as easily allowed to enter working memory. For the learned irrelevance condition, higher flexibility might still be beneficial to clear out the representation of the previous target color but should also come with a higher distractibility by the novel target color of the distractor. Increased stability should in contrast not be negatively affected by the higher stickiness of the previous target color, because even if it is not yet cleared out of working memory, the color is not presented after the switch and does therefore not need an updated stimulus-response-mapping. Furthermore, a higher updating threshold also might be more beneficial in this condition than in the perseverance condition, because it shields working memory representations from interference by the novel stimulus color of the distractor.

By calculating a difference score between the conditions, we do not focus on differences between individuals within conditions, but on the relative costs and benefits of the conditions within individuals, eliminating variance due to more general individual performance differences. Higher positive difference scores, caused by relatively higher switch costs in the learned irrelevance and lower switch costs in the perseveration condition, should be associated with higher flexibility. Lower or even negative scores should in turn be associated with higher stability.

### Procedure

Participants first gave their written informed consent to take part in the study, confirmed they did not consume alcohol, nicotine, or caffeine within the last 12 h, and confirmed that they were not pregnant using a standard test (10 mIU/ml human chorionic gonadotropin hCG, VEDA.LAB, Alençon cedex, France). After a light standardized breakfast, they received a capsule either containing sulpiride (200 mg) or a placebo, and completed various personality questionnaires as well as a test of fluid intelligence. Approximately 1 hour after administration of the capsule participants started with the 3-back task, followed by the switching task. After several other tasks, including an experimental manipulation of positive emotions (between groups) and a test of cognitive flexibility (Wacker, 2018), participants completed a standardized postexperimental interview, received their financial compensation, and left the lab after approximately 5 hours.

### Statistical Analysis

We computed a multivariate multiple linear model in order to analyze the effects of condition (placebo vs. sulpiride), agentic extraversion, and their interactions on the performance in the switching and in the 3-back task within one analysis. The significance level was defined as α = 0.05. By conducting a multivariate model with an overall *F*-test first, the following univariate F-tests, which are only performed if the overall F-test is significant, are protected against an inflation of the overall error rate (Rencher, 2002).

To analyze the three main outcomes (3-back speed and accuracy, switch cost difference score) in more detail and to compare our findings with previously reported results on the relationship between agentic extraversion and EFs, we afterwards conducted multiple linear models for each of the reported outcomes with a step-wise method.

In a first step, we calculated one model for each outcome with the same predictors as in the multivariate model. In a second step, we added the respective other outcomes and their interactions with condition into the models to analyze whether the effects were specific to the respective task or could be explained by shared performance variance. We then analyzed within-condition correlations (partialing out potential confounding variables) as additional effect size measures on the significant interaction effects of each regression model. We did not include 3-back speed as a covariate into the model with 3-back accuracy as the outcome (and vice versa), because 3-back speed and 3-back accuracy are based on the same task and therefore are not informative concerning the specificity of effects.

Both n-back and switching tasks have several other outcomes, which could be potentially used as performance measures. We therefore report additional analyses using signal detection measures (*d’* and *C*) as well as a measure of RT variability for the 3-back task and variations of switch cost measures (per condition, overall mean switch costs, other intervals around the switch) for the switching task.

## Results

### Preliminary Analyses

#### Side Effects and Blindness to Condition

None of the participants reported any adverse side effects in response to the pill they received. As part of a postexperimental interview, the answers of participants in a forced choice question whether or not they thought they had been given the drug were independent of their experimental condition (χ^2^ (1, *N* = 80) = 0.74, *p* = 0.39). The self-reported certainty of the 17 participants (on a scale from 0-100%) who guessed that they had been given the drug did not differ significantly between the sulpiride (*M* = 62.73, *SD* = 19.79) and the placebo (*M* = 58.33, *SD* = 29.78) condition (*t*(15) = 0.37, *p* = 0.719). None of the participants who guessed correctly about having received sulpiride reported to be 100% sure. It therefore can be concluded that participants were blind to the experimental conditions.

#### Preexisting Differences Between Conditions

We did not find any preexisting significant differences between experimental conditions for age (*t*(90) = 0.893, *p* = 0.374), fluid intelligence score (*t*(90) = −0.037, *p =* 0.971), or any of the NEO scales in self- or partner-ratings (all *p*s > 0.10, except the partner rating of agreeableness, *t*(89) = −1.90, *p =* 0.06).

#### Data Exclusion Based on Task Performance

Inspection of reaction time and accuracy data from the switching and the 3-back task showed that ten participants had to be excluded from the analyses due to incompliance, difficulties with understanding the task, or technical difficulties. In the 3-back task, two participants failed to respond in more than 35% of all 3-back trials, which indicated that they did not comply or had difficulties with the task instructions (while all other participants nearly always gave a response, *M* = 99.07%, *SD* = 0.02). Four other participants failed to react within their individual response window in more than 25% of all trials (>30 trials), leaving it questionable whether the individual latency criterion had the intended effect on their performance compared to the other participants (mean number of trials with “slow”-feedback for the other participants was *M* = 5.67, *SD* = 3.68). In the switching task, three participants had very high error rates in all pre switch trials of the switching task in one of the conditions (>92%), suggesting a technical problem, and the error rate of one other participant indicated that she did not comply or had difficulties with the task instructions (28.75%; error rates of all other participants were much lower, *M* = 5.79%, *SD* = 3.24). All cases with invalid data on at least one dependent variable were excluded. This resulted in a final sample of *n* = 82 participants (40 in the placebo and 42 in the sulpiride condition). Descriptive statistics for the NEO-PI-R scale and the two executive functioning tasks for the analysis sample are displayed in Table 1. In order to examine whether or how much our exclusion decisions influenced the results of our main analysis, we performed the main analysis with alternative exclusion decisions in which we (1) did not exclude any participants, (2) did not exclude any participants but transformed the data to achieve normality despite the included outliers, or (3) only made exclusions based on one of the reasons listed above, but not on the respective others. The results of the alternative analyses, although less pronounced, displayed the same pattern and did not yield any additional information (*p*-range for the hypothesized interaction effect: 0.022-0.105). All results of these analyses are displayed in Table 3 in the Supplement.

**Table 1:** Means and Standard Deviations for the Big Five Domains and for Performance Measures of the Executive Functioning Tasks

		Condition		
		Placebo	Sulpiride	Total
		*M* (*SD*)	*M* (*SD*)	*M* (*SD*)
NEO-PI-R Scale	Neuroticism	91.92 (18.3)	94.27 (16.7)	93.1 (17.5)
	Extraversion	117.9 (13.8)	122.7 (16.5)	120.4 (15.33)
	Agentic extraversion	18.2 (2.8)	18.1 (4.1)	18.1 (3.5)
	Affiliative extraversion	22.0 (3.1)	23.0 (3.4)	22.5 (3.3)
	Openness	122.6 (11.3)	123.6 (15.2)	123.1 (13.4)
	Agreeableness	116.3 (11.4)	121.7 (14.3)	119.1 (13.2)
	Conscientiousness	125.3 (16.0)	122.2 (20.7)	123.7 (18.5)
Dependent Variables	3-back accuracy	60.4 (17.1)	58.8 (15.9)	59.6 (16.5)
	3-back speed	697.4 (146.3)	683.4 (132.0)	690.3 (138.4)
	Switching task:			
	Δ switch costs	24.8 (76.6)	-3.5 (84.5)	10.3 (81.5)
	Perseveration	29.9 (72.8)	41.7 (71.3)	36.0 (71.9)
	Learned irrelevance	53.5 (68.3)	38.1 (74.9)	45.7 (71.7)
	Total switch costs	41.9(58.8)	39.9 (59.7)	40.9 (58.9)
n		40	42	82

Δ switch costs = switch cost difference (switch costs *perseveration* minus switch costs *learned irrelevance*).

#### Reliability

The switch cost difference showed clearly unsatisfactory reliability (Cronbach’s α = 0.22), whereas the reliability for the switch costs per block was higher but still unsatisfactory (Cronbach’s α = 0.41 for learned irrelevance, and Cronbach´s α = 0.47 for perseveration). Reliability was equally low when not taking five but ten trials around the switch into account when calculating switch costs, indicating that the low reliability is not only due to the limited amount of trials included per block. The original goal of the switching task was, like for most other cognitive-behavioral measures, to minimize between-participant variability and maximize within-subject effects (e.g., of reward or positive affect; Dreisbach & Goschke, 2004; Müller, Dreisbach, Goschke, et al., 2007). The undeniably low reliability therefore may not only be a sign of high error variance, but also of relatively low between-subject variability, complicating its application in individual differences research (Hedge et al., 2018). We will further address this issue in the discussion section.

We also calculated the split-half reliability (corrected with the Spearman-Brown prophecy formula) for both measures of the 3-back task, which was excellent for 3-back speed (*Rel* = 0.91) and good for 3-back accuracy (*Rel* = 0.83).

### Main Analysis

We calculated an analysis of variance for a multivariate multiple linear regression model with the predictors condition (sulpiride vs. placebo), agentic extraversion, and the interaction between condition and agentic extraversion. We analyzed the effects of the predictors on the three outcome measures *3-back speed*, *3-back accuracy*, and the *switch cost difference* (Table 2). We found a significant main effect for agentic extraversion (ω^2^ = 0.12). As expected, multivariate cognitive performance was also significantly explained by an interaction between condition and agentic extraversion (ω^2^ = 0.13). In order to understand how shared performance variance among the two tasks influenced task performance, and if the predictors also account for task-specific variance, we next analyzed each of the three outcomes separately with two-step linear models (Table 2).

**Table 2:** Multivariate und Univariate Multiple Linear Regression Models

		Multivariate Model			Univariate Models						
						3-back accuracy		3-back speed		Δ switch costs	
Parameter		*df*	*Wilks’ λ*	*approx. F*	*df*	*F*_1_	*F*_2_	*F*_1_	*F*_2_	*F*_1_	*F*_2_
Step 1	Condition (placebo vs. sulpiride)	3	0.96	1.04	1	0.20	0.01	0.09	0.07	2.89	3.30
	aE	3	0.85	4.53**	1	5.06*	1.62	2.14	2.01	5.40*	1.83
	Condition * aE	3	0.86	4.15**	1	8.32**	4.46*	0.01	0.04	4.12*	2.76
	Error	76			78						
Step 2	3-back accuracy				1						4.32*
	3-back speed				1						0.22
	Δ switch costs				1		5.41*		0.02		
	Condition * 3-back accuracy				1						7.72**
	Condition * 3-back speed				1						0.09
	Condition * Δ switch costs				1		8.72**		0.58		
	Error				74						

Note. *N* 82; *F1* F-statistics for the step 1 model; *F2* F-statistics for the step 2 model; *aE* Agentic Extraversion; *Δ switch costs* switch cost difference (switch costs perseveration minus switch costs learned irrelevance). Agentic Extraversion was centered within condition

*p < .05

**p < .01

***p < .001

#### 3-back Task: Accuracy

**Fig. 1:**
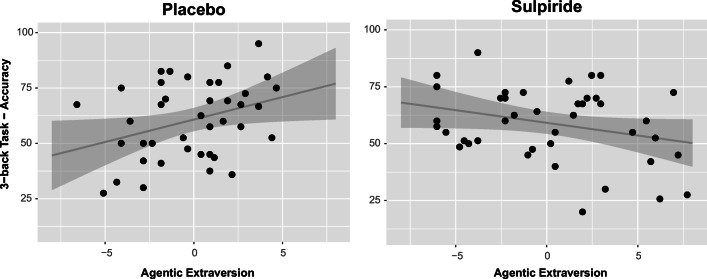
Scatterplot showing the correlations of agentic extraversion (centered within condition) and accuracy in the 3-back task (in %) with fitted linear regression lines and 95% confidence intervals

For 3-back accuracy, we found a significant main effect for agentic extraversion (ω^2^ = 0.05) and a significant interaction between condition and agentic extraversion (ω^2^ = 0.08) in step 1. The interaction effect was due to a positive correlation between agentic extraversion and 3-back accuracy in the placebo condition (*r*(38) = 0.33, *p* = 0.03) and a correlation in the opposite direction in the sulpiride condition (*r*(40) = −0.28, *p* = 0.07; difference: *z* = 2.71, *p* = 0.006; Fig. 1).

It is still unclear at this point whether the significant interaction between condition and agentic extraversion specifically explains performance in 3-back accuracy or whether performance differences are shared among the two tasks. We therefore entered the switch cost difference as a covariate into the model (main effect and first-order interaction with condition) to perform step 2. The interaction between condition and agentic extraversion was attenuated but remained significant (ω^2^ = 0.04), while the main effect for agentic extraversion disappeared almost completely (ω^2^ = 0.01). Additionally, we found a significant main effect for the switch cost difference (ω^2^ = 0.05) and a significant interaction effect between condition and the switch cost difference (ω^2^ = 0.09). We examined the interaction effects more closely with partial correlations between agentic extraversion and 3-back accuracy, controlling for the switch cost difference. The correlation decreased somewhat in the placebo condition (*r*(38) = 0.20, *p* = 0.22), and remained nearly unchanged in the sulpiride condition (*r*(40) = −0.30, *p* = 0.054; difference: *z* = 2.25, *p* = 0.026). The interaction effect between condition and the switch cost difference was carried by a positive partial correlation (controlling for agentic extraversion) between 3-back accuracy and the switch cost difference in the placebo condition (*r*(38) = 0.35, *p* = 0.028). This correlation tended to be reversed for the sulpiride condition (*r*(40) = −0.28, *p* = 0.072, difference: *z* = 2.88, *p* = 0.004).

The significant interaction effect between condition and the switch cost difference on 3-back accuracy, as well as the numeric decrease in the placebo group’s significant correlation between agentic extraversion and 3-back accuracy after partialing out the switch cost difference, indicate that the two tasks share some variance. However, neither the effect of agentic extraversion (in interaction with condition) on 3-back accuracy in the linear model, nor their correlation (within conditions), disappeared completely after including the switch cost difference as a covariate. This indicates that the significant interaction between agentic extraversion and condition can partly be ascribed to performance variance shared with the switching task, and partly to variance specific to the 3-back task. The tendency for correlations per condition in opposite directions between 3-back accuracy and the switch cost difference may further suggest a differential sensitivity of the two tasks to sulpiride; however, given that this effect was unexpected it should be regarded as preliminary.

#### 3-back Task: Speed

For 3-back speed, we found no main or interaction effects in either step 1 or 2. Shorter reaction times in correct target trials of the 3-back task were associated with higher agentic extraversion (*r*(80) = −0.27, *p* = 0.013), although this association was not pronounced enough in order have any meaningful effect in the model (*p* = 0.15, ω^2^ = 0.01).

#### Switching Task: Switch Cost Difference

For the switch cost difference, we found a significant main effect for agentic extraversion (ω^2^ = 0.05) and a significant interaction between condition and agentic extraversion (ω^2^ = 0.04) in step 1. The interaction effect was carried by an association between the switch cost difference and agentic extraversion in the placebo condition, indicating that higher flexibility was correlated with higher agentic extraversion (*r*(38) = 0.38, *p* = 0.014). This association was completely absent in the sulpiride condition (*r*(40) = −0.03, *p =* 0.86), but the difference between correlations for the placebo versus the sulpiride condition failed to reach significance (*z* = 1.84, *p =* 0.066). The correlation between agentic extraversion and the switch cost difference in the placebo condition was equally driven by the two task conditions (perseveration: *r*(37) = −0.197, *p* = 0.228; learned irrelevance: *r*(38) = 0.205, *p* = 0.205).

In order to clarify whether the significant interaction between condition and agentic extraversion specifically explains performance in the switching task, or whether performance differences are shared among the two tasks, we entered the two measures of the 3-back task as covariates into the model (main effects and first-order interactions) in step 2. The interaction effect between condition and agentic extraversion of step 1 was somewhat attenuated (*p* = 0.10, ω^2^ = 0.02). The same was found for the main effect for agentic extraversion (*p* = 0.18, ω^2^ = 0.01). Besides a significant main effect for 3-back accuracy (ω^2^ = 0.04), we found a significant interaction effect between condition and 3-back accuracy (ω^2^ = 0.08), mirroring the significant (reverse) interaction effect of condition and the switch cost difference on 3-back accuracy. This interaction effect was carried by the above-mentioned positive partial correlation (controlling for agentic extraversion) between 3-back accuracy and the switch cost difference in the placebo condition, which tended to be reversed for the sulpiride condition. Similar to the pattern on the models for 3-back accuracy, this pattern again suggests that the two tasks share some variance, and that this shared performance variance contributes at least partly to the significant interaction effect between condition and agentic extraversion.

The two conditions of the switching task are designed to capture different cognitive processes, and these processes might be differentially associated with 3-back accuracy. We therefore exploratively investigated correlations between switch costs per condition (learned irrelevance and perseverance) and 3-back accuracy. While there were no significant correlations in the perseverance condition (see Supplement Table 1), we found a clear pattern in the learned irrelevance condition: The positive correlation between 3-back accuracy and learned irrelevance switch costs was quite pronounced in the placebo condition (*r*(39) = 0.408, *p* = 0.009), but absent in the sulpiride condition (*r*(39) = −0.165, *p* = 0.297, difference: *z* = 2.54, *p* = 0.011). This suggests that the learned irrelevance condition drives the association with 3-back accuracy. All pairwise correlations per condition for all task performance indices (including all alternative performance indices) can be found in Table 1 of the Supplement.

### Additional Analyses

#### Specificity to Agentic Extraversion

To investigate whether our findings were specific to agentic extraversion and not explained by other covariates (alternative extraversion measures, all other NEO scales, fluid intelligence or body weight), we recalculated the main multivariate multiple linear regression model separately including one covariate per model (all results are displayed in Table 4 of the Supplement). The previously reported significant effects of agentic extraversion remained significant after entering each covariate, respectively, whereas none of the covariates had significant effects. The exception was, as expected, the model in which we replaced NEO agentic extraversion with an agentic extraversion measure from the MPQ, which displayed the same pattern of significant effects as the main model. Interestingly, the model in which we replaced NEO agentic extraversion with the complete NEO extraversion scale did not reveal any significant effects. Based on these findings, we concluded that our findings were indeed specific to agentic extraversion.

#### Alternative Performance Indices Derived From the Switching and 3-back Task

As several other performance indices exist for both tasks, we investigated the effects of condition, agentic extraversion, and their interaction on the most commonly reported ones in separate univariate linear regression models in Table 2 in the Supplement (alternative indices for the 3-back task: d’, C, total accuracy, RT variability; for the switching task: switch cost difference for ±10 trials around the switch, switch costs for both conditions separately, total switch costs). Reliabilities for the alternative performance indices are depicted in Table 1 in the Supplement. Regarding the alternative 3-back performance indices, we only found a small main effect of agentic extraversion and an interaction effect of condition and agentic extraversion for response bias C. Higher agentic extraversion was associated with a more liberal response bias in the placebo condition (*r*(38) = 0.340, *p* = 0.032), and with a more conservative response bias in the sulpiride condition (*r*(40) = −0.324, *p* = 0.036; difference: *z* = 2.93, *p* = 0.003). This observation fits previous speculations (Wacker et al., 2006) that individual differences in extraversion are rather associated with *how* a task is done and not *how well* it is done. Regarding the alternative indices for the switching task, we only found significant effects on the switch cost difference for ten trials around the switch with a slightly weaker interaction effect (*p* = 0.071), demonstrating that results were not substantially altered by the number of trials around the switch. The fact that none of the other alternative indices revealed any significant effects might further indicate that the focus on the relative costs and benefits in the two conditions within individuals is necessary to investigate predictors of individual differences.

## Discussion

The goals of the current study were to investigate (1) whether there is an association between agentic extraversion and EFs measured with either the 3-back and/or the switching task, (2) whether this association is sensitive to manipulations of brain DA, and (3) whether the effects are due to shared or specific task variance in the EF tasks. We found the expected significant interaction effect between agentic extraversion and DA drug condition on EF task performance in a multivariate model. Thus, a pharmacological manipulation of DA D2 receptors only had an effect on EF task performance in interaction with agentic extraversion, but not alone. Furthermore, the univariate analyses showed the expected interaction effect between agentic extraversion and condition on the switch cost difference and on 3-back accuracy, but not on 3-back speed. After controlling for the respective other EF task performance, both interaction effects were somewhat attenuated.

### Agentic Extraversion is Associated with Task Performance in the Placebo Condition

We found agentic extraversion to be positively associated with both updating performance measured via 3-back accuracy and cognitive flexibility measured via the switch cost difference in the placebo condition. The positive association between extraversion and 3-back performance matches previous findings with updating tasks (Campbell et al., 2011; Lieberman & Rosenthal, 2001; Wacker et al., 2006). Our additional analysis finding that agentic extraversion was associated with a more liberal response bias in the placebo condition further supports the idea that extraversion-related differences in task performance might not only be due to differences in ability, but in the way the task is performed (Wacker et al., 2006).

Because there are only few existing studies on the association between agentic extraversion and tasks targeting cognitive flexibility, and they revealed mixed results, we view the positive relationship between agentic extraversion and switching performance found in this study with caution. The current study is the first to apply the switching task in a context of extraversion-related individual differences and a direct comparison between this and other measures for cognitive flexibility seems to be missing. It therefore remains unclear whether the current and other tasks measure the underlying construct of cognitive flexibility to a similar extent.

Contrary to Wacker et al. (2006), we did not find any association for 3-back speed, but only for 3-back accuracy. This could indicate that participants of the current study applied a different strategy, potentially because they were only confronted with the 3-back but not easier task versions (0-, 1-, and 2-back). However, given the lack of convergence of the results observed here and by Wacker et al. (2006), the current findings should be regarded as preliminary.

### Dopamine Modulates the Relationship Between Agentic Extraversion and Task Performance

**Fig. 2:**
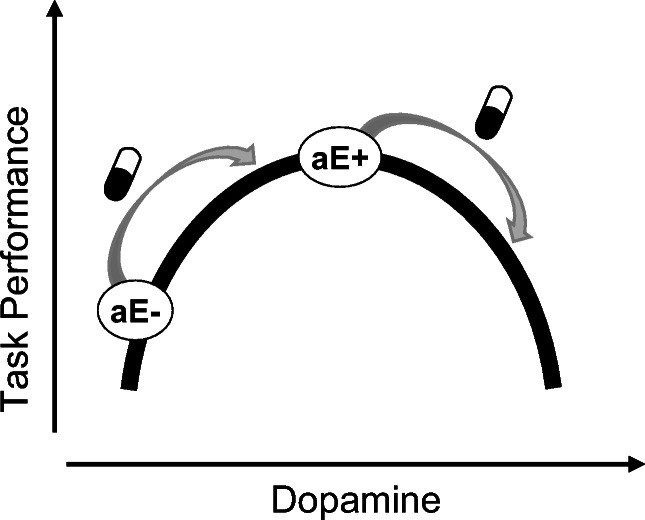
Inverted-U shaped model depicts the relationship between baseline dopamine and performance in executive functioning tasks for individuals low (aE-) or high (aE+) in agentic extraversion. The arrows illustrate how the association between agentic extraversion and task performance is attenuated (or even reversed) under sulpiride

The association between agentic extraversion and performance in both tasks was sensitive to sulpiride, which mainly affects DA receptors in the striatum (Sigala et al., 1991). The current data do not speak to the neurophysiological mechanisms underlying this effect but invite the speculation that extraversion-related dopaminergic differences in the striatum might have caused the differential response to sulpiride. More specifically, higher agentic extraversion might be associated with better updating performance and higher flexibility because of higher striatal DA activation, leading to a lower updating threshold (Berse et al., 2014). When striatal DA activation is further enhanced via sulpiride, the updating threshold might be lowered, even to a no longer functional level, which is behaviorally reflected in distractibility. In contrast, the same dopaminergic manipulation might optimize performance for less extraverted people (who start with a higher updating threshold due to lower baseline striatal DA) by beneficially lowering the updating threshold without reaching the point of distractibility (Fig. 2). Our data partly support this notion because we found a matching pattern for the switch cost difference in the switching task and for accuracy (but not speed) in the 3-back task. Although this explanation would match our data, there are several alternative explanations. Individuals high versus low in agentic extraversion might not differ in striatal DA activation, but might be differentially sensitive towards changes in the balance between D1 and D2 receptor activation, or more generally to changes in DA levels, because of individual differences in other neural structures within the corticostriatal loop (Doll & Frank, 2009). Sulpiride might further not only have an effect on cognitive control itself, but also on the motivation to exert it (Cools et al., 2019).

The two tasks seemed to differ in their sensitivities to sulpiride, as the placebo group´s positive association between agentic extraversion and flexibility in the switching task was significantly lower in the sulpiride condition but was not reversed like in the 3-back task.

Considering that DA alterations can have a variety of effects across different tasks and domains (Floresco, 2013), it remains unclear whether such differential sulpiride effects on the two tasks should be ascribed to differential neural mechanisms of the EFs they supposedly measure. Alternatively, the two tasks might recruit mostly the same dopaminergic mechanisms, but the DA level for optimal performance might vary between tasks. In any case, our unexpected findings of differential effects of sulpiride on performance in the two tasks should therefore be regarded as preliminary, but hopefully give rise to further investigation.

### Relationship Between the 3-back Task and the Switching Task

The pattern of significant interaction effects of condition and agentic extraversion in both tasks, and their attenuation after the inclusion of the respective other EF task performance measure, suggests that these interaction effects can be at least partly ascribed to shared task variance. This refers either to the unity of EF tasks, i.e., a shared executive component which is relevant for both tasks, or to task-impurity, i.e., other systematic variance of processes needed for the task, for example visual processing, number/letter processing or manual motor skills. As the switching task is based on difference scores, which would theoretically alleviate task-impurity (Kessler et al., 2017), we suggest that shared variance might at least partly be ascribed to the unity of EFs. From this would follow that agentic extraversion might be associated with a unitary component of EF variance common to most EF tasks rather than being separately associated with specific EFs. This has the more general implication that future research on personality-EF associations should be cautious with statements of specific associations if only one EF task was investigated.

The placebo group’s positive association between accuracy in the 3-back task and flexibility in the switching task seemed to be driven mainly by the switching task’s learned irrelevance condition. Higher 3-back accuracy was clearly associated with higher switch costs in the learned irrelevance condition (assumed to measure distractibility), while there was no association for the perseverance condition (assumed to measure stickiness of no longer relevant representations). This may be taken to indicate that stickiness might be less important in the 3-back task, whereas individual differences in the updating threshold are relevant for both tasks. A lower threshold might be advantageous for 3-back accuracy, as the 3-back task requires rapid updating of new information, but disadvantageous for switch costs in the learned irrelevance condition, as this condition is (partly) constructed to capture distractibility due to a low updating threshold. However, because the interpretation of the association between 3-back accuracy and switch costs in the learned irrelevance condition is complicated by its unexpected sensitivity to sulpiride, it should be regarded as preliminary.

### Limitations

Some methodological limitations have to be considered. The homogeneous sample of healthy young females who all were in a heterosexual partnership and took hormonal contraception was chosen to minimize variance but limits the generalizability of our results. Statistical power was sufficient for general cognitive effects but might have not been optimal for the investigation of associations between task performance and personality as prior studies might have overestimated effect sizes. Furthermore, we did not control for the intake method of hormonal contraception or for the amount of estradiol derivates, which varies among contraceptive medications. Although all participants received the same amount of sulpiride (200 mg), it seems unlikely that a variation in the relative dose per kilogram body weight was associated with performance differences because we did not find a significant effect of body weight as a covariate. However, prior studies reported an inverted-U relationship between DA and working memory functions (Cools & D’Esposito, 2011), as well as an inverted-U relationship between extraversion and EEG theta activity (Chavanon et al., 2013), which were both demonstrated with the help of varying doses of DA agonists and antagonists. A systematic investigation of (non-linear) dose-dependent effects of DA agonists and antagonists could provide further insight into their effects on the relationship between extraversion and EFs. Because the plasma concentration of sulpiride further varies over time with a peak after 1 to 6 hours (Mauri et al., 1996), there is a small possibility that plasma concentration varied systematically between the two tasks, which were presented in a fixed order after one another. We assume that this had little impact on performance because, relative to the length of the tasks, the variation in peak plasma concentration is quite large.

Additionally, the behavioral patterns we found do not necessarily have to be directly associated with altered striatal DA or the balance between prefrontal and striatal DA. The current pharmacological alteration of striatal DA might have various complex effects within the corticostriatal loop, which can indirectly affect other systems. EFs also are not exclusively regulated by DA but are partly also sensitive to noradrenergic manipulations (Arnsten, 2011). A more detailed understanding of the neural areas and processes regulating task performance could be facilitated with the help of neuroimaging during task completion and/or with computational models (Chatham et al., 2011; Herd et al., 2014). The latter could be fruitful for a deeper understanding of the neurobiological basis of extraversion by simulating individual differences within these models.

Unclarity regarding construct validity and reliability, especially of the switching task, also needs to be considered. First, despite repeated use of the switching task in (mostly) cognitive research (Müller, Dreisbach, Brocke, et al., 2007; Müller, Dreisbach, Goschke, et al., 2007) to our knowledge we are the first to report reliability estimates for the switching task. It should be noted that low reliability does not necessarily equal high measurement variance but can also be caused by low between-subject variability. Because experimental effects across all individuals are more pronounced when between-subject variability is low, a low reliability might even be seen as an unintended prerequisite of a successful cognitive task, which complicates the translation to the investigation of individual differences (Hedge et al., 2018). In any case, the low reliability observed here clearly limits the conclusions to be drawn from measured individual differences in the switching task and future studies using indicators for shifting with higher psychometric quality are needed to confirm the current observations. It might be a fruitful approach to maximize between-subject variability and to minimize measurement variance by applying more frequent switches and/or more task blocks. Between-subject variability could be increased by making the task more demanding, e.g., with a latency criterion, in order to have higher error rates.

Second, while the majority of studies on the n-back task report accuracy measures (Karr et al., 2018), a prior study reporting an association with extraversion found effects in reaction times but not in accuracy (Wacker et al., 2006). Taken together, it would be interesting to investigate whether the tasks and their respective measures used here actually measure the same constructs as other EF tasks and their respective measures. Investigating the interaction of extraversion and a dopaminergic manipulation with latent EFs, e.g., within the unity/diversity framework (Friedman & Miyake, 2017), would help to clarify matters of validity and task impurity.

### Conclusions

In this study, our goal was to elucidate the link between EFs and agentic extraversion by using more than one EF task to compare shared and task-specific variance in EF tasks. We found agentic extraversion in the placebo condition to be associated with performance in both the 3-back task and the switching task. Furthermore, the results from our additional analyses were likewise compatible with the interpretation that the association between each individual task and agentic extraversion can at least partly be ascribed to shared variance among the two tasks. Thus, previous investigations on extraversion-EF associations may have overestimated their specificity to a certain EF, and future research may consider the unity of EFs, as well as task impurity of EF tasks, for the investigation of third-variable associations. Furthermore, we found the extraversion-EF associations to be sensitive to a dopaminergic manipulation, which extends prior findings indicating a functional interplay or overlap of the neuronal systems regulating EFs and agentic extraversion. Future pharmacological studies using more than two EF tasks with satisfactory reliability in conjunction with a latent variable approach in large samples are necessary to bolster the current conclusions and to further connect the fields of personality research, cognitive psychology, and neuroscience.

## Supplementary Information

ESM 1 (DOCX 42.3 kb)

## Footnotes

1 In this paper, we will use the term *cognitive flexibility* as a general concept and the term shifting when referring to the more task- and process-oriented *shifting factor*. The term *switching* will only be used when referring to the particular switching task that was used in this study.
