# Supplementary material for: The Selective Dopamine D2 Blocker Sulpiride Modulates the Relationship Between Agentic Extraversion and Executive Functions
Source: Cogn Affect Behav Neurosci. 2021 Apr 2;21(4):852–67. doi: 10.3758/s13415-021-00887-9 (PMC8354875; doi:10.3758/s13415-021-00887-9)
Supplement: Supplementary file 1 — (DOCX 42.3 kb) [file 13415_2021_887_MOESM1_ESM.docx]

**Supplement**

Table 1

*Correlations and reliabilities of all dependent variables and alternative performance indices within condition*

|  | Measure | 1 | 2 | 3 | 4 | 5 | 6 |  | 7 | 8 | 9 | 10 | 11 |
| --- | --- | --- | --- | --- | --- | --- | --- | --- | --- | --- | --- | --- | --- |
| 3-back measures | |  |  |  |  |  |  |  |  |  |  |  |  |
| 1. | target accuracy |  | .23 | .71** | .70** | .51** | -.06 |  | -.26 | -.22 | -.16 | .14 | -.02 |
| 2. | target speed | .21 |  | .18 | .15 | .13 | .42** |  | -.15 | -.06 | .04 | .21 | .15 |
| 3. | d‘ | .72** | .11 |  | -.01 | .88** | -.07 |  | -.07 | -.10 | -.12 | -.04 | -.09 |
| 4. | C | .69** | .19 | .01 |  | -.17 | -.02 |  | -.30 | -.20 | -.12 | .23 | .07 |
| 5. | total accuracy | .58** | .07 | .91** | -.11 |  | .05 |  | -.02 | -.08 | -.06 | -.03 | -.05 |
| 6. | RT variability | .09 | .58** | .15 | -.02 | .16 |  |  | -.18 | .00 | -.12 | .09 | -.02 |
|  |  |  |  |  |  |  |  |  |  |  |  |  |  |
| switching task measures | |  |  |  |  |  |  |  |  |  |  |  |  |
| 7. | Δ switch costs (+/- 5 trials) | .43** | -.06 | .46** | .15 | .26 | -.06 |  |  | .74** | .61** | -.55** | .05 |
| 8. | Δ switch costs (+/- 10 trials) | .30 | .02 | .29 | .13 | .09 | .04 |  | .78** |  | .43** | -.43** | -.02 |
| 9. | switch costs learned irrelevance | .41** | .08 | .48** | .09 | .38* | -.01 |  | .51** | .53** |  | .33* | .83** |
| 10. | switch costs perseverance | -.07 | .16 | -.03 | -.08 | .09 | .11 |  | -.58** | -.32* | .41* |  | .81** |
| 11. | total switch costs | .19 | .15 | .27 | .00 | .27 | .06 |  | -.06 | .14 | .83** | .85** |  |
|  | Reliability | .83 | .91 | .75 | .86 | .74 | .57 |  | .22 | .21 | .41 | .47 | .58 |

*Note.* *N* = 82. Correlations below the diagonal refer to the placebo group, above to the sulpiride group. For all 3-back task measures reliability is indicated by the split half reliability (corrected with the Spearman-Brown prophecy formula), for all switching task measures by Cronbach´s α.
* *p* < .05 ** *p* < .01

Table 2

*Alternative analyses of 3-back and switching task performance with univariate multiple linear regression models*

|  |  | *F* | | | | | |
| --- | --- | --- | --- | --- | --- | --- | --- |
| Alternative performance measure | | condition | | agentic extraversion | | condition x agentic extraversion | |
| 3-back task | |  |  |  |  |  |  |
|  | d' | 0.01 |  | 0.81 |  | 1.02 |  |
|  | C | 0.28 |  | 5.17 | * | 9.45 | ** |
|  | total accuracy | 0.43 |  | 0.38 |  | 0.01 |  |
|  | RT variability | 0.82 |  | 0.10 |  | 0.00 |  |
| switching task | |  |  |  |  |  |  |
|  | Δ switch costs (+/- 10 trials) | 2.81 |  | 4.09 | * | 3.34 |  |
|  | switch costs learned irrelevance | 0.96 |  | 1.48 |  | 1.93 |  |
|  | switch costs perseverance | 0.70 |  | 1.51 |  | 0.58 |  |
|  | total switch costs | 0.01 |  | 0.00 |  | 0.17 |  |
|  |  |  |  |  |  |  |  |

*Note.* *N* = 82. Agentic extraversion was centered within condition. RT variability was Box-Cox transformed to normalize the distribution. As these additional analyses only serve a descriptive purpose, we did not control the family-wise error rate. Accuracy in the switching task was not analyzed because the error rates were too low (*M* = 5.79%, *SD* = 3.24), creating ceiling effects.

* *p* < .05 ** *p* < .01

Table 3

*Results of multivariate multiple linear regression models after different exclusion decisions*

|  |  |  | | Condition | | | Agentic extraversion | | | Condition x Agentic extraversion | | |
| --- | --- | --- | --- | --- | --- | --- | --- | --- | --- | --- | --- | --- |
| Exclusion criterion | | Analysis n | df | Wilks’ λ | *approx. F* | *p* | Wilks’ λ | *approx. F* | *p* | Wilks’ λ | *approx. F* | *p* |
| 1. | No Exclusion | 92 | 3, 83 | 0.95 | 1.36 | 0.260 | 0.91 | 2.62 | 0.056 | 0.93 | 2.10 | 0.105 |
| 2. | Transformation only | 92 | 3, 83 | 0.95 | 1.32 | 0.274 | 0.90 | 3.00 | 0.035* | 0.928 | 2.16 | 0.099 |
| 3-back task | |  |  |  |  |  |  |  |  |  |  |  |
| 3a. | No response | 88 | 3, 80 | 0.96 | 1.08 | 0.362 | 0.87 | 3.83 | 0.013* | 0.908 | 2.70 | 0.051 |
| 3b. | Too slow response | 90 | 3, 82 | 0.96 | 1.16 | 0.331 | 0.90 | 3.10 | 0.031* | 0.89 | 3.38 | 0.022* |
| Switching task | |  |  |  |  |  |  |  |  |  |  |  |
| 3c. | High error rates before switch | 88 | 3, 82 | 0.95 | 1.43 | 0.239 | 0.91 | 2.85 | 0.042* | 0.92 | 2.29 | 0.084 |

*Note.* Each model has three response variables (3-back speed, 3-back accuracy and switch cost difference). Agentic extraversion was centered within condition. Exclusion criteria are explained in more detail in the ‘preliminary analyses’ section of the main document. **1.** No participants excluded. **2.** 3-back accuracy and 3-back speed were Box-Cox transformed, Δ switch costs were Yeo-Johnson transformed, in order to achieve normality. **3a.** Participants who failed to respond in more than 35% of all 3‑back trials. **3b.** Participants who failed to react within their individual response window in more than 25% of all trials (> 30 trials). **3c.** Participants who had high error rates in all pre switch trials of the switching task in one of the conditions.

* *p* < .05 ** *p* < .01

Table 4

*Results of multivariate multiple linear regression model after inclusion of covariates*

|  |  | Correlation  with aE | | *F* | | | | | | | | | | | |  | | |
| --- | --- | --- | --- | --- | --- | --- | --- | --- | --- | --- | --- | --- | --- | --- | --- | --- | --- | --- |
| Included covariate | |  |  | condition | | | aE | | covariate | | | condition x  aE | | | condition x covariate | | | |
| Extraversion scales | |  |  |  |  |  | |  | |  |  |  |  |  | | |  |  |
|  | MPQ aE | .58 | *** | 0.95 |  |  | |  | | 4.51 | ** |  |  | 3.06 | | | * |  |
|  | NEO extraversion |  |  | 0.97 |  |  | |  | | 0.44 |  |  |  | 1.35 | | |  |  |
|  | NEO affiliative extraversion | .36 | ** | 1.06 |  | 5.04 | | ** | | 0.96 |  | 3.55 | * | 0.71 | | |  |  |
| Other NEO scales | |  |  |  |  |  | |  | |  |  |  |  |  | | |  |  |
|  | Neuroticism | -.37 | *** | 1.01 |  | 4.71 | | ** | | 0.53 |  | 3.60 | * | 0.47 | | |  |  |
|  | Openness | .01 |  | 1.02 |  | 4.67 | | ** | | 0.50 |  | 4.35 | ** | 0.52 | | |  |  |
|  | Agreeableness | -.12 |  | 1.04 |  | 3.24 | | * | | 2.06 |  | 3.70 | * | 1.70 | | |  |  |
|  | Conscientiousness | .37 | *** | 1.03 |  | 3.03 | | * | | 0.97 |  | 2.78 | * | 1.94 | | |  |  |
| Further covariates | |  |  |  |  |  | |  | |  |  |  |  |  | | |  |  |
|  | CFT fluid intelligence | .01 |  | 1.07 |  | 5.25 | | ** | | 0.80 |  | 4.79 | ** | 1.10 | | |  |  |
|  | Body weight | -.14 |  | 1.01 |  | 3.70 | | * | | 0.82 |  | 3.70 | * | 0.14 | | |  |  |

*Note.* *N* = 82. aE = NEO agentic extraversion. Each row represents one model. All covariates are centered within condition. As these additional analyses only serve a descriptive purpose, we did not control the family-wise error rate. As NEO extraversion and MPQ agentic extraversion were investigated as replacements for aE, aE was not included as covariate into the respective models due to large overlaps in variance. The correlation between NEO extraversion and aE is not listed because the scales overlap.

* *p* < .05 ** *p* < .01
